# Supplementary figures and images for: Revealing the dynamics of ultrarelativistic non-equilibrium many-electron systems with phase space tomography
Source: Sci Rep. 2023 Mar 21;13:4618. doi: 10.1038/s41598-023-31196-5 (PMC10030633; doi:10.1038/s41598-023-31196-5)

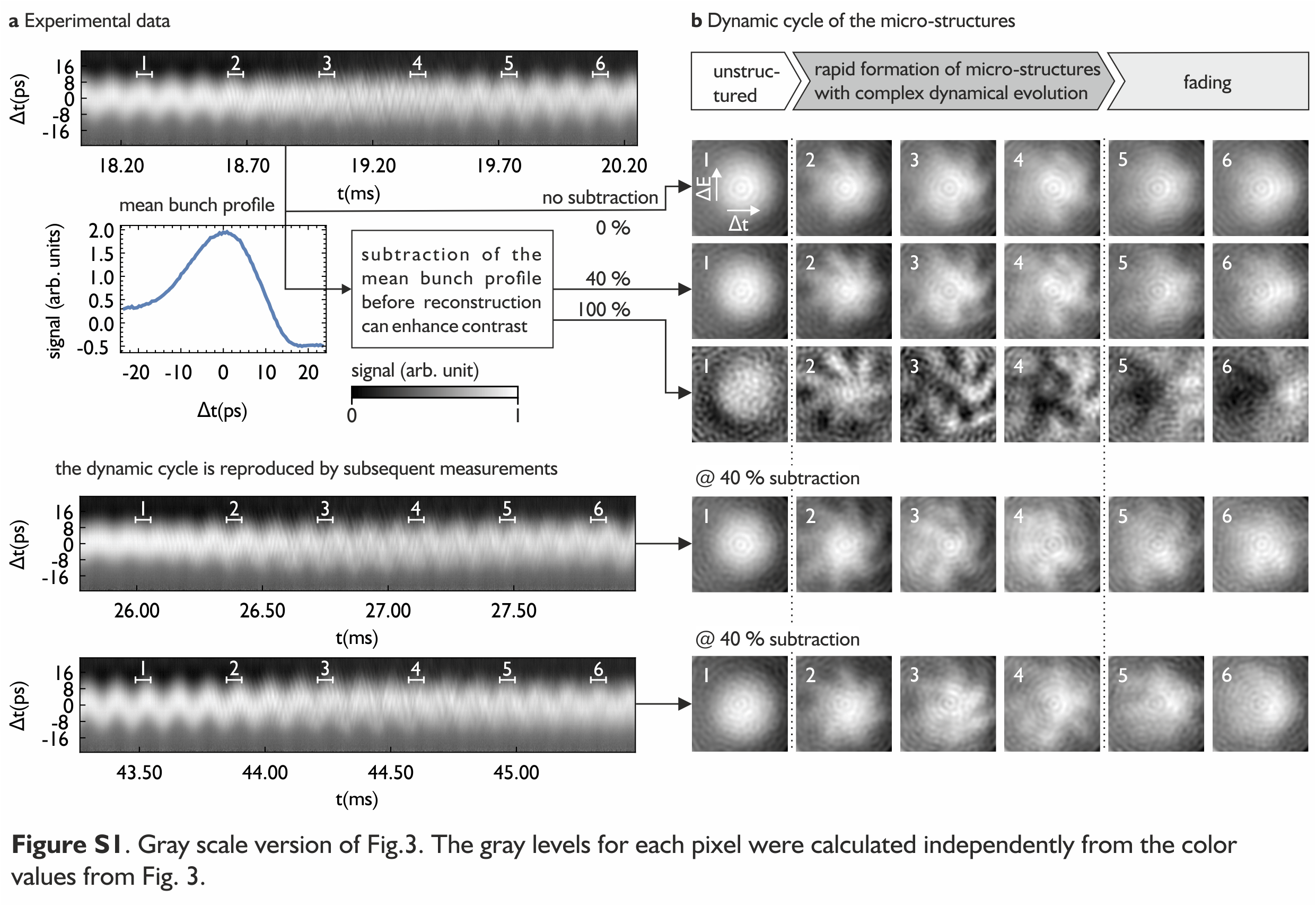

Supplement: Supplementary file 1 — Supplementary Information 1. [file 41598_2023_31196_MOESM1_ESM.png]

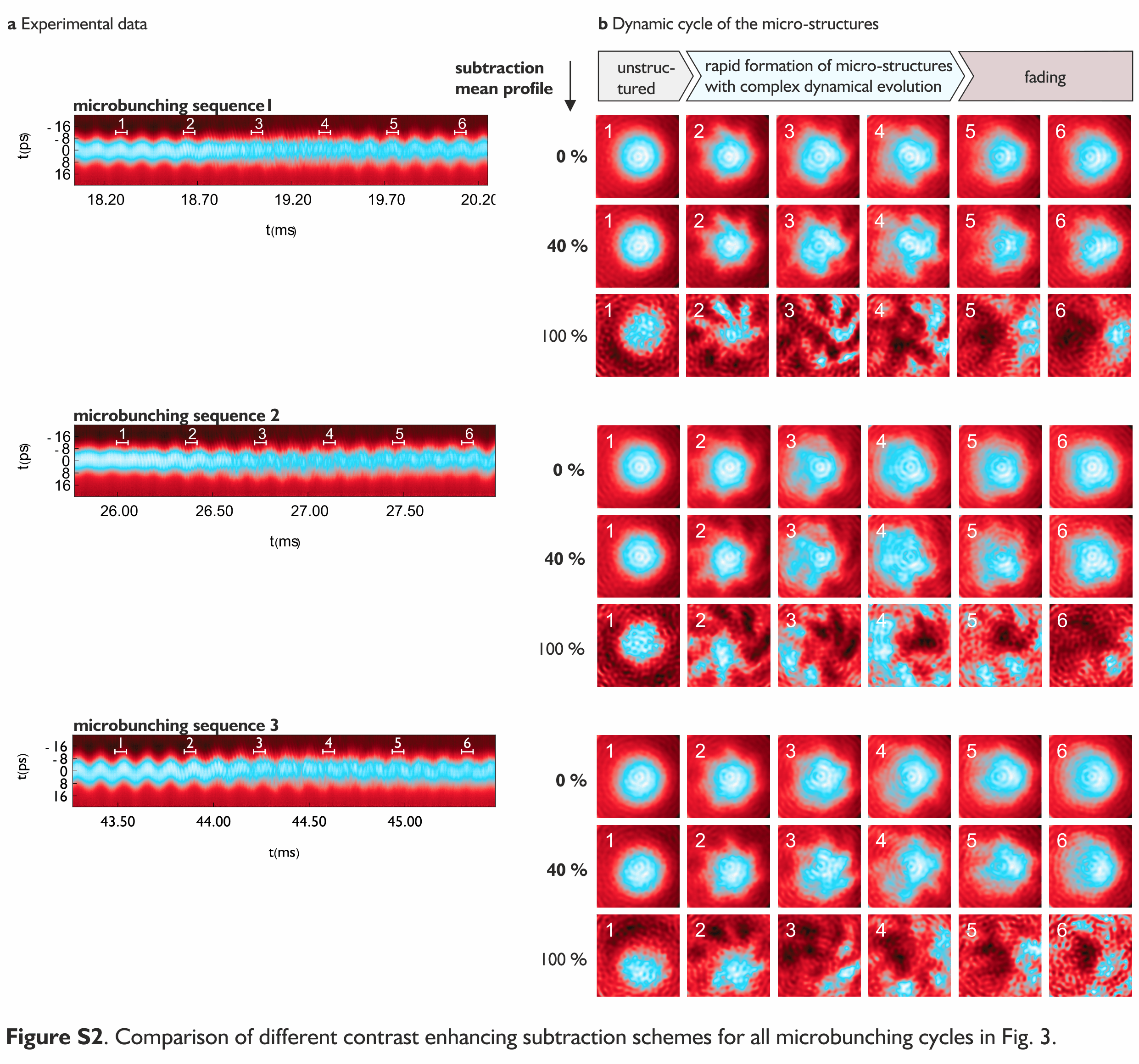

Supplement: Supplementary file 2 — Supplementary Information 2. [file 41598_2023_31196_MOESM2_ESM.png]
